# Human iPS cell-derived engineered heart tissue does not affect ventricular arrhythmias in a guinea pig cryo-injury model

**DOI:** 10.1038/s41598-019-46409-z

**Published:** 2019-07-08

**Authors:** Simon Pecha, Kaja Yorgan, Matti Röhl, Birgit Geertz, Arne Hansen, Florian Weinberger, Susanne Sehner, Heimo Ehmke, Hermann Reichenspurner, Thomas Eschenhagen, Alexander Peter Schwoerer

**Affiliations:** 1 DZHK (German Center for Cardiovascular Research, partner site Hamburg/Kiel/Lübeck), Department of Cardiovascular Surgery, Cardiovascular Research Center, University Medical Center Hamburg-Eppendorf, Hamburg, Germany; 2 DZHK (German Centre for Cardiovascular Research, partner site Hamburg/Kiel/Lübeck), Department of Experimental Pharmacology and Toxicology, Cardiovascular Research Center, University Medical Center Hamburg-Eppendorf, Hamburg, Germany; 3 DZHK (German Centre for Cardiovascular Research, partner site Hamburg/Kiel/Lübeck), Institute of Cellular and Integrative Physiology, Cardiovascular Research Center, University Medical Center Hamburg-Eppendorf, Hamburg, Germany; 4 Department of Medical Biometry and Epidemiology, University Medical Center Hamburg-Eppendorf, Hamburg, Germany

**Keywords:** Regeneration, Cardiac regeneration

## Abstract

Human iPSC-derived engineered heart tissue (hEHT) has been used to remuscularize injured hearts in a guinea pig infarction model. While beneficial effects on cardiac remodeling have been demonstrated, the arrhythmogenic potential of hEHTs is a major concern. We investigated whether hiPSC-derived hEHTs increase the incidence of ventricular arrhythmias. HEHTs were created from human iPSC-derived cardiomyocytes and endothelial cells. Left-ventricular cryo-injury was induced in guinea pigs (n = 37) and telemetry sensors for continuous ECG monitoring were implanted. 7 days following the cryo-injury, hEHTs or cell-free constructs were transplanted into the surviving animals (n = 15 and n = 9). ECGs were recorded over the following 28 days. 10 hEHT animals and 8 control animals survived the observation period and were included in the final analysis. After implantation of hEHTs or cell-free constructs, ventricular arrhythmias (premature ventricular contractions, couplets, triplets and non-sustained ventricular tachycardia) were observed in animals of both groups. The fraction of animals with the respective arrhythmias as well as the rate of arrhythmic events did not differ between groups. Following hEHT implantation, no clinically relevant sustained ventricular tachycardia or ventricular fibrillation was detected. Our telemetric data provides first evidence for the electrical safety of human iPSC-derived EHTs in this experimental model, thereby supporting further development of this approach.

## Introduction

Heart failure is increasing in an aging population^1^. Besides heart transplantation, there is no curative treatment option for those patients. Due to organ-donor shortage, new, organ-independent treatment options are needed. Recent achievements in mechanical circulatory support are promising, but especially in the long-term run this approach is limited by its side effects like infections, pump thrombosis and bleeding complications^2^. Due to the limited endogenous regeneration capacity of human cardiomyocytes, regenerative therapies might become a future option for treatment of end-stage heart failure^3,4^. Different methods including stem cell-based cell injection therapies or tissue engineering approaches have been described^4–7^.

Nowadays, human induced pluripotent stem cells (hiPSC), as well as human embryonic stem cells (hESC) are the most promising sources which can be effectively differentiated to spontaneously beating human cardiomyocytes^8^. The beneficial effects of cardiomyocyte transplantation on left-ventricular function and cardiac remodeling has been repeatedly demonstrated in animal models of heart failure^7,9–14^. The potential impact of transplanted cardiomyocytes on ventricular electrophysiology, however, is a major concern. Proarrhythmicity would represent a relevant hurdle to the successful implementation of these new therapeutic strategies^9,10^. Amongst others, reentry by incomplete electrical coupling of the graft, automaticity of spontaneously active cells, or triggered activity of single (immature) cardiomyocytes within the graft could induce singular or even sustained arrhythmias. On the other hand, a reduction of scar formation in the infarction area by the transplanted tissue could beneficially affect electrophysiology and reduce the number of ventricular arrhythmias. Only few studies have directly addressed the effects of injected hiPSC and hESC on electrical coupling and remodeling *in vivo* and have inconsistently reported pro- and antiarrhythmic effects in different small and large animal models^9,11,12,15^. To date, no data on arrhythmic effects of implantation of three-dimensional hiPSC-derived human engineered heart tissue (hEHT) is available.

We have recently reported that hEHT from hiPSC-derived cardiomyocytes can remuscularize injured hearts and improve left-ventricular function in a guinea pig model^7^. We were able to directly demonstrate electrical coupling of the grafts in at least some of the hEHT animals^7^. The arrhythmogenic consequences of the hEHT transplantation in this context of incomplete coupling, was not assessed in our previous study. Based on the amount of the predominantly proarrhythmic concepts and on the recent literature using other modes of cardiomyocyte transplantation and animal models, we hypothesized that hiPSC-derived hEHT implantation increases the incidence of ventricular arrhythmias following myocardial infarction in this small animal model. To determine the arrhythmogenic risk of hEHT transplantation, we therefore implanted telemetric devices in a subset of animals of the initial cohort from our previously published study^7^. ECG was recorded over a period of 28 days following implantation of hEHT (n = 20) or control grafts (n = 17). Here, we present the data obtained during continuous ECG analysis.

## Results

**Figure 1:**
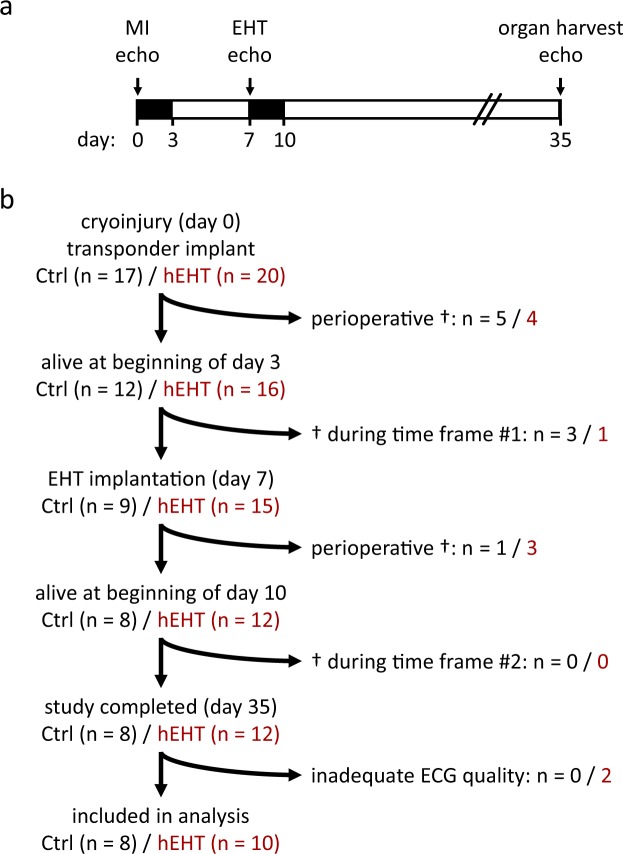
Study design. (**a**) Telemetric ECG devices were implanted in the same procedure as cryo-injury (MI) was performed on day 0. At day 7, all surviving animals received either hEHT or control constructs. The perioperative periods were defined as the first 48 h following each intervention and were excluded from ECG analysis (black time frames). ECG analysis was performed following cryo-injury (day 3–day 6) and following graft implantation (day 10–day 35). Echocardiography (Echo) was performed at days 0, 7 and 35. On day 35, organs were harvested for histological examination. (**b**) Study flow chart, including animal numbers and details on animal deaths (†).

Figure 1 gives an overview of the study design (Fig. 1a) and illustrates the animal numbers and dropouts during the study (Fig. 1b). Myocardial cryo-injury was induced in 37 animals which were randomized to the control group (n = 17) or to the hEHT group (n = 20). Control or hEHT grafts were implanted at day 7 following cryo-injury (Fig. 1a). As previously reported^7^, the large myocardial injury and the perioperative burden during cryo-injury and graft implantation resulted in a high overall mortality within the first 8 days after cryo-injury (53% in the control group and 40% in the hEHT group, Fig. 1b). Notably, in the time frame relevant for the ECG analysis of the graft impact (day 10–day 35 following cryo-injury), no animal of either group died. Following completion of the study, two animals of the hEHT subgroup had to be excluded from the study due to an insufficient quality of the ECG recordings. Therefore, 8 control and 10 hEHT animals completed the study protocol and were included in the following analysis.

**Figure 2:**
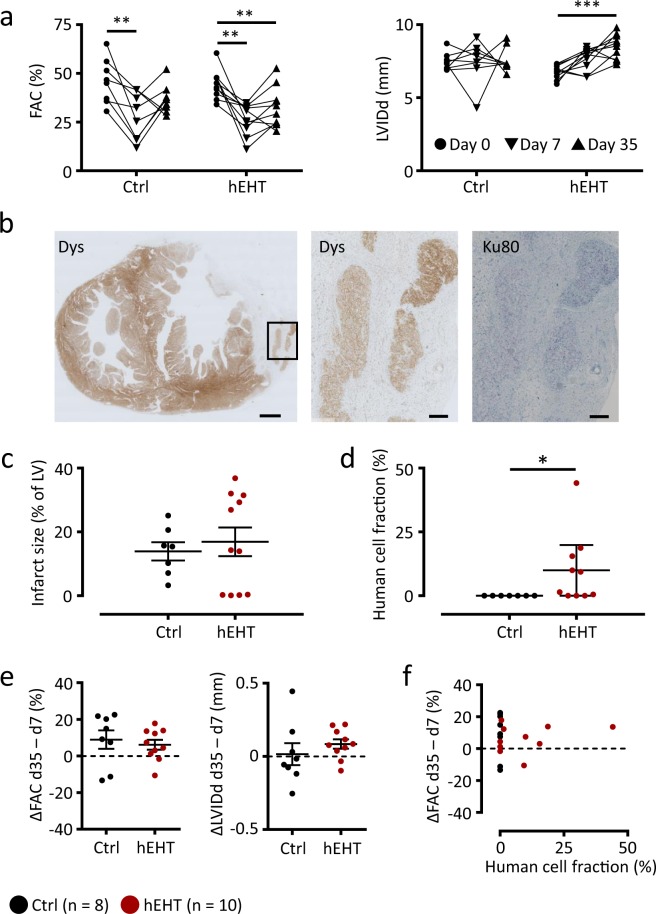
Functional parameters, infarct size and human cell fraction. (**a**) Fractional area change (FAC) and left-ventricular inner diastolic diameter (LVIDd) in the control and hEHT group immediately before cryo-injury (day 0, ●), before graft implantation (day 7 after injury, ▼) and at the end of the observation period (day 35 after injury, ▲). (**b**) Representative transversal section at low magnification (left panel) and high magnification of the area indicated by the box (middle and right panel). Sections were stained for dystrophin (Dys) and Ku80 to validate human origin. Scale bars represent 1 mm (left panel) and 200 µm (middle and right panel). (**c**) Infarct size (% of left ventricle) and (**d**) mean human cell fraction (%) determined within the grafts. (**e**) Effect of graft implantation on fractional area change (FAC) and left-ventricular inner diastolic diameter (LVIDd). Values were calculated as differences between day 7 and day 35 following cryo-injury (ΔFAC and ΔLVIDd). (**f**) Correlation analysis between ΔFAC (ΔLVIDd) and human cell fraction. Data are given (**a**) as before-after plots and as individual data points with lines and error bars denoting (**c**) mean ± SEM and (**d**) median ± interquartile range, respectively. (**e**) Lines and error bars denote mean ± SEM. Results were analyzed with (**a**) an one-way ANOVA followed by multiple comparisons using a Tukey test with multiplicity adjusted P values and with (**c**,**e**) a parametric and (**d**) with an unparametric (Mann-Whitney) unpaired Student’s t-test. *P < 0.05, **P < 0.01, ***P < 0.001. Ctrl, animals implanted with cell-free fibrin constructs (n = 8); hEHT, animals with implanted hEHTs (n = 10).

Cryo-injury was performed with a nitrogen-cooled aluminum probe and resulted in a significant reduction in left-ventricular function with no significant differences between the hEHT and the control group. Fractional area change (FAC) decreased significantly between day 0 and day 7 while left-ventricular geometry (e.g. diastolic left ventricular inner diameter, LVIDd) was not affected (Fig. 2a). Also, heart rates, physical activity and the health scores of the animals did not differ between the two groups during the time frame directly following myocardial injury (day 3 to day 6, data not shown). As a global readout, this implicates a similar overall health status, and sympathetic tone, in hEHT and control animals. Thus, no significant difference in the myocardial infarction between the control and the hEHT group could be found prior to graft implantation.

One week following cryo-injury, two hEHTs or one cell-free fibrin construct (with the same size as two hEHTs, negative control) were sutured onto the guinea pig heart (day 7). At the end of the observation period (day 35), a large transmural myocardial injury could be verified histologically in all cryo-injured hearts (Fig. 2b). Infarct sizes did not differ between both groups (Fig. 2c). Using dystrophin staining, compact muscle islands could be demonstrated histologically and immunohistochemically within the scar area in the hEHT group. The human origin of the grafts was verified by immunohistochemical staining for human Ku80 (Fig. 2b,d). While in most animals surviving human cells were detectable, the fraction of human cells was low in some of the hEHT animals (Fig. 2d). This could relate to the selection of the analyzed sections or might indicate ischemic cell death or rejection of the transplanted human cells in this xenogenic setting.

**Figure 3:**
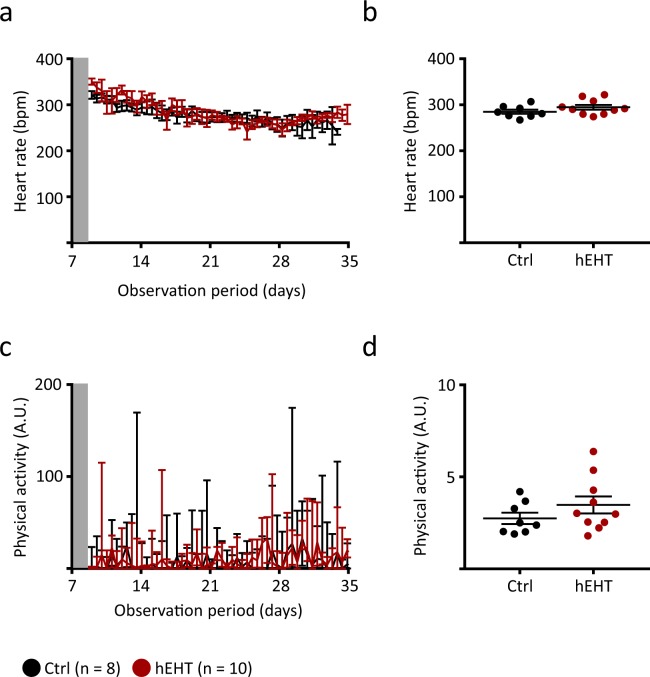
Heart rate regulation and physical activity. (**a**) Heart rate of control and hEHT animals following implantation of the hEHTs (day 10–day 35, data are averaged over 12 h and are given as mean ± SEM). (**b**) Average heart rates following implantation of the EHTs (day 10–day 35, lines and error bars denote mean ± SEM). (**c**) Physical activity of control and hEHT animals following implantation of the hEHTs (day 10–day 35, data are averaged over 12 h and are given as median ± interquartile range, arbitrary units, A.U.). (**d**) Average physical activity (arbitrary units, A.U., day 10–day 35, lines denote median and whiskers represent min to max). (**a**–**d**) Results were not statistically significantly different between the groups using a parametric (**b**) and an unparametric (**d**, Mann-Whitney test) unpaired Student’s t-test. Grey zones (**a**,**c**) indicate postoperative period recordings (48 h) which were excluded from analysis. Ctrl, animals implanted with cell-free fibrin constructs (n = 8); hEHT, animals with implanted hEHTs (n = 10).

Echocardiographic analysis at day 35 did not reveal significant differences between both groups with respect to FAC and LVIDd (Fig. 2a). In most animals of both groups, the FAC did not further decrease and the LVIDd remained unaffected (ΔFAC and ΔLVIDd, Fig. 2e). There was no correlation between the human cell fraction and the change in FAC (Fig. 2f). Furthermore, heart rate regulation (Fig. 3a,b) as well as physical activity (Fig. 3c,d) were similar between both groups, again implicating a comparable overall health status following graft implantation (day 10 to day 35).

**Figure 4:**
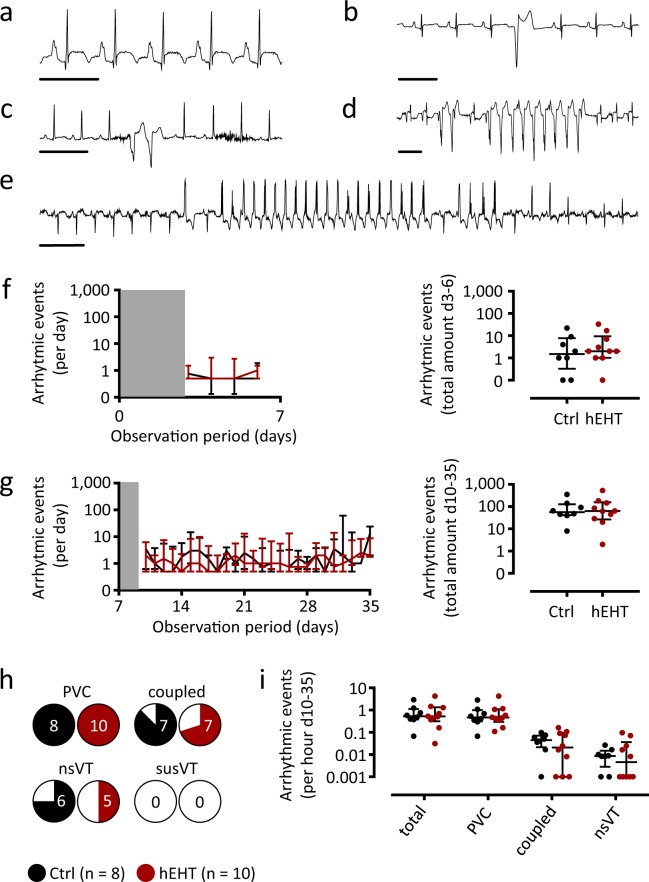
ECG analysis. Representative ECGs showing (**a**) a normal ECG in sinus rhythm, (**b**) a premature ventricular contraction (PVC), (**c**) a couplet, (**d**) a non-sustained ventricular tachycardia (nsVT) and (**e**) a sustained VT (susVT). Scale bars represent 250 ms. (**f**,**g**) Time course of average arrhythmic events per day (left panels) and total amount of ventricular arrhythmias (right panels) following cryo-injury (**f**, day 3–day 6) and following implantation of hEHTs (**g**, day 10–day 35). Data are visualized using median ± interquartile range. Grey zones indicate postoperative period recordings (48 h) following induction of MI and graft implantation which were excluded from analysis. Statistical differences between both groups were calculated using a mixed effects linear regression for longitudinal data with animals as cluster variable and the day by group interaction if significant; else both main effects (left panels). Potential differences for total amount of arrhythmias were compared using negative binominal regression with the groups as predictor (right panels). (**h**) Fraction of control and hEHT animals in which the different subtypes of ventricular arrhythmias could be detected following hEHT implantation (day 10–day 35). The occurrence of any arrhythmic event was analyzed using mixed effects logistic regression with animals as cluster variable and the interaction between types of arrhythmic events and group if significant; else both main effects. (**i**) Incidence of arrhythmias by subclass following hEHT implantation (day 10–day 35). Data are visualized using individual data points and median ± interquartile range. Potential differences were calculated using mixed effects negative binominal regression with animals as cluster variable and the interaction between types of arrythmias and group if significant; else both main effects. Ctrl, animals implanted with cell-free fibrin constructs (n = 8); hEHT, animals with implanted hEHTs (n = 10).

**Figure 5:**
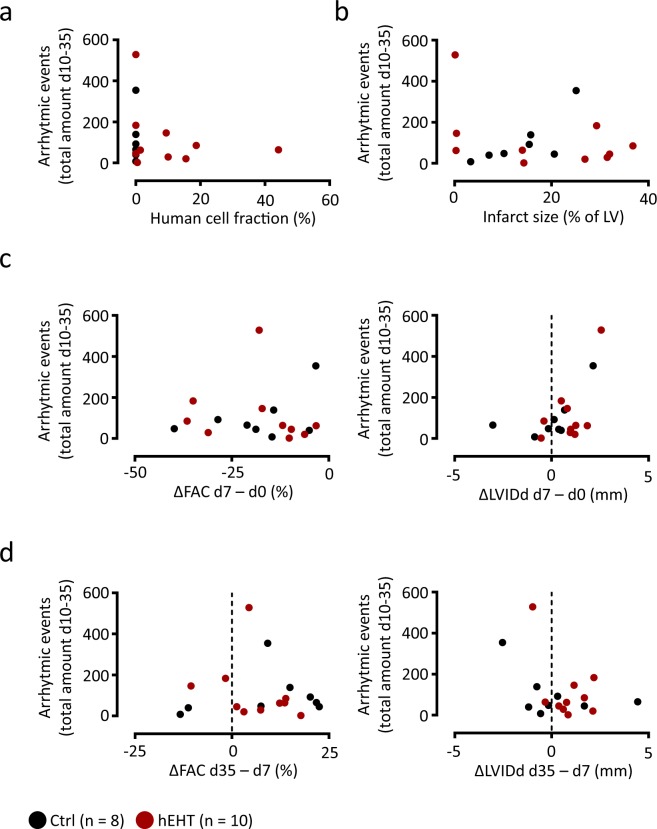
Arrhythmic burden. Plots illustrating the relation between the total number of ventricular arrhythmias between day 10 and day 35 and (**a**) the human cell fraction, (**b**) the histologically confirmed infarct size, (**c**) the effect of the myocardial injury on fractional area change (ΔFAC day 7–day 0) and left ventricular inner diastolic diameter (ΔLVIDd day 7–day 0), and (**d**) the effect of the implanted grafts on FAC (ΔFAC day 35–day 7) and LVIDd (ΔLVIDd day 35–day 7). Data are calculated based on the data visualized in Figs 2 and 4.

ECG analysis of the observation periods following cryo-injury (day 3–day 6) and following graft implantation (day 10–day 35) revealed sinus rhythm in all animals with several types of ventricular arrhythmias, e.g. premature ventricular contractions (PVCs), coupled beats (couplets and triplets), non-sustained ventricular tachycardia (nsVT) as well as one episode of sustained ventricular tachycardia (susVT, Fig. 4a–e). In none of the animals, a ventricular fibrillation (VFib) was observed. In the time frame between myocardial injury and hEHT implantation (day 3–day 6), the incidence of ventricular arrhythmias was similar between the hEHT and control group (Fig. 4f). Here, the only sustained ventricular tachycardia was detected in one control animal Fig. 4e). Following hEHT implantation, the arrhythmic burden (ventricular arrhythmias per day) was similarly distributed over the observation period (day 10–day 35) and between the groups (Fig. 4g). PVCs could be detected in all hEHT and in all control animals, and most animals also displayed coupled beats and nsVTs (Fig. 4h). When calculated for the whole observation period, neither the incidence, nor time course of any type of ventricular arrhythmias (Fig. 4g), nor the fraction of animals with specific arrhythmias (Fig. 4h), nor the incidence of the different arrhythmic subtypes (Fig. 4i) was significantly different between hEHT and control animals. Furthermore, the absolute number of ventricular arrhythmias following graft implantation was independent of the fraction of surviving human cells (Fig. 5a) and of the histologically confirmed infarct sizes (Fig. 5b). Based on functional (e.g. ΔFAC) and geometrical parameters (e.g. ΔLVIDd), the arrhythmic event rate did not correlate with the effect of the cryo-injury (Fig. 5c) and with the remodeling following graft implantation (Fig. 5d).

## Discussion

Previously, we have demonstrated beneficial effects of hEHT transplantation on cardiac remodeling and ventricular function in a guinea pig cryo-injury model^7^. The current study was performed to assess the risk of arrhythmia induction by hEHTs in this experimental model. Overall, we detected a similar number of ventricular arrhythmias (PVCs, coupled beats and non-sustained ventricular tachycardia) between hEHT and control animals. Furthermore, no episodes of sustained ventricular tachycardia or ventricular fibrillation were detected following implantation of the grafts. We, therefore, provide the first evidence that heart tissue engineered from human induced pluripotent stem cells does not induce arrhythmias in this cryo-injury model.

Currently, there are mainly two approaches to remuscularize injured hearts with hiPSC-derived cardiomyocytes. Either via direct intramyocardial injection^9,11,15^ or via the generation of cardiac patches for transplantation^7,12,16,17^. While the beneficial effects of these approaches on ventricular function have repetitively been demonstrated, their impact on the ventricular electrophysiology remains a major concern. The potential to induce severe ventricular arrhythmias would clearly impede the translation of these techniques into patients. Only few studies have directly addressed the effects of hiPSC- and hESC-derived cardiomyocyte grafts on the electrical remodeling *in vivo*^9,11,12,15^. Notably, these studies used different applications, different animal models and reported inconsistent results. Most previously published studies were performed using intramyocardial cardiomyocyte injection^9,11,15^. Of these, two studies in non-human primates reported a proarrhythmic potential of hiPSC and hESC grafts^11,15^ and one demonstrated an antiarrhythmic effect by hESC grafts in guinea pigs^9^. In a porcine model of myocardial infarction, hiPSC grafts did not affect the incidence of ventricular arrhythmias^12^. The current study was performed in guinea pigs since this species combines experimental feasibility with a cardiac electrophysiology which is in several aspects similar to the human situation. In particular, ionic currents which may be relevant for arrhythmia induction (e.g. delayed K^+^ currents) can also be found in human hEHTs^13,18^. Moreover, the physiological heart rate of guinea pigs (~300 bpm) is closer to the intrinsic beating rate of the human hEHTs (60–120 bpm) than that of rats or mice.

In an injured heart, an increase of left-ventricular function by implanted grafts could improve electrophysiological properties of the host myocardium and, thereby, reduce the arrhythmic burden^14^. This concept has been proposed by Shiba *et al*. who have reported an antiarrhythmic effect of intramyocardial injection of pluripotent stem cell-derived cardiomyocytes in a guinea pig model^9^. Using hEHTs generated from hIPS-derived cardiomyocytes, we have previously also demonstrated positive effects on systolic function, organ geometry and electrical remodeling following cryo-injury in guinea pigs. In particular, ventricular action potential duration was shortened and conduction velocity was increased in hearts that had received hEHTs^7^. In spite of these beneficial effects, the current study does not document an overall antiarrhythmic potency and, therefore, seems to be in contrast to the concept proposed by Shiba *et al*.^9^. However, in the animals included in the current study, we did not see a statistically significant improvement of ventricular function and geometry 35 days after cryo-injury. While in some animals receiving hEHTs left-ventricular function strongly improved, left-ventricular function was unaffected in others. This is in line with our previously published study in which we could demonstrate an systolic improvement by hEHT grafts in spite of a large biological variability using a higher number of animals as in the current study^7^. An inadequate electrical coupling between the grafts and the viable myocardium could also reduce the improvement of systolic function, thereby reducing the beneficial effects on left-ventricular electrophysiology. Due to the study protocol, cellular coupling was not directly investigated in the current subset of animals. Therefore, we cannot provide evidence for or against electrical coupling in these specific animals. However, also in animals with pronounced improvement of left-ventricular function (which argues for electrical coupling) no changes in arrhythmias were observed (ΔFAC, Fig. 5d). The absence of a statistically significant improvement of left-ventricular function in the current study is, therefore, most likely caused by biological heterogeneity (e.g. graft size, graft integration) and variances inherent to technical aspects (e.g. echocardiography in infarcted animals). Interestingly, Shiba *et al*. reported only a very modest antiarrhythmic effect under baseline conditions and mainly demonstrated an antiarrhythmic potential upon electrophysiological stimulation^9^. Under unstimulated physiological conditions which may be more clinically relevant, our current study is, therefore, well in line with Shiba *et al*.^9^ and does not support the notion that hEHTs exert antiarrhythmic effects.

In contrast, transplanted cardiomyocytes could promote arrhythmias at least via three, potentially synergistic, mechanisms. (i) Automaticity: although regular differentiation protocols result in cardiomyocytes that mainly demonstrate a ventricular phenotype and exhibit similar electrophysiological properties as ventricular human cardiomyocytes^18–20^, these cells are immature and demonstrate spontaneous diastolic depolarization^18,21,22^. (ii) Afterdepolarizations: human iPSc-derived cardiomyocytes show a lower repolarization reserve^18^ and afterdepolarizations occur regularly^23^. Transplantation probably even favors conditions (e.g. due to ischemia) that promote afterdepolarizations. (iii) Reentry mechanisms: there is evidence for electrical graft-host coupling after cardiac repair. However, coupling was less frequent after transplantation in the scar when compared to healthy myocardium^9,23^ or occurred only in a subset of animals^7^. Transplantation of either cells or patches regularly results in isolated cardiomyocyte grafts that are separated from the host myocardium by scar tissue representing anatomical pathways for reentry mechanisms. Furthermore, conduction velocity could be slower in the graft than in the host tissue^9^ also favoring reentry mechanisms. A recent study in non-human primates provided first evidence that arrhythmias more likely evolve from ectopic activity (automaticity or afterdepolarizations) of the transplanted cells than from conduction abnormalities^24^.

It is, therefore, remarkable that despite of these potential proarrhythmic effects, and despite the absence of an antiarrhythmic effect associated with an improvement of left-ventricular function, the incidence of ventricular arrhythmias in the current study was not increased by hEHT implantation. One could argue that the hEHTs did not induce a significant number of arrhythmias due to a low percentage of surviving human cardiomyocytes. However, the arrhythmic burden did not correlate with the human cell fraction (Fig. 5a). Interestingly, we observed a notable variability in detected human cell fraction in hEHT animals. This itself could be related to ischemic cell death, wash-out after transplantation, rejection or, technical limitations. Even though vascularization by host-derived vessels can be observed^7^, there certainly is a phase post-transplantation during which the cells are poorly supported with oxygen. Although, the hIPSC derived cardiomyocytes might have a high ischemia tolerance due to their immaturity, there might be ischemic cell death in this phase. Furthermore, in this xenogenic setting, rejection of the transplanted human cells could occur. Although, we did not observe histological evidence for rejection 28 days after implantation, serial histologic investigations in the early phase after transplantation, are necessary to exclude early graft rejection. In this context, a closer surveillance of blood immunosuppression levels should also allow a more reliable immunosuppression. Finally, the high variability of detected human cell fractions could also be a consequence of the limitations in a small animal model. Repeated thoracotomies can lead to pleural adhesions which severely aggravate EHT transplantation and might eventually impair cell survival. Taken together, however, our data does not support the hypothesis that the absence of proarrhythmogeneity is a consequence of a low human cell fraction.

At present, the finding of our study seems to be in contrast to the two independent studies that reported proarrhythmic effects of transplanted cardiomyocytes in large animal models^11,15^. This discrepancy might result from differences in intrinsic heart rates (with lower heart rates favoring automaticity and/or triggered activity) or in geometry (with larger heart volumes that could provide a substrate for reentry mechanisms). It could, however, also be a consequence of the application pathway. Most studies reporting proarrhythmic effects were based on cell injection, while antiarrhythmic effects or no effect on ventricular arrhythmias were reported following patches/EHTs. Finally, electrical integration in the host myocardium is not well understood after patch transplantation. Although our previous study provided evidence for electrical coupling in a small subset of animals, two recent studies did not confirm this finding in a rat model^25,26^. In contrast, they reported that cardiac patches did not electrically couple after transplantation. In this context, it will be interesting to study, to which degree the differences between the intrinsic beating rates of *in vitro* cultured hIPSCs (~60 bpm) and *in vivo* host myocardium affects cell coupling. The lower heart rates of larger animals (or humans) could facilitate electrical cell integration, thereby preventing arrhythmic events^12^. The experiences with direct cell injection, however, currently rather point in the other direction. A slower heart rate and the larger heart favor proarrhythmic mechanisms that are masked in a small animal model^27^. These mechanisms may become relevant even with marginal electrical coupling – and may outweigh the potentially beneficial effects of the lower heart rates on electrical integration. As currently no robust data of large animal models is available, we cannot exclude the possibility of proarrhythmic effects by hEHTs in larger animal models. Further electrophysiological studies in small and large animal models will be necessary to evaluate electrical coupling.

In conclusion, the current work used human iPSC-derived EHT transplanted on a guinea pig cryo-injury model to remuscularize injured hearts. Over an observation period of 28 days following implantation, the incidence of ventricular arrhythmias (regardless of the subtype) was not affected by hEHTs. Thereby, the study provides first evidence that transplanted cardiomyocytes in hEHTs do not increase the arrhythmic burden. This finding is important for the safety considerations and encourages further development of this approach for myocardial regeneration.

## Methods

### Study Protocol

The current study has been performed in a subgroup of animals that has been included for general phenotyping in the paper by Weinberger *et al*.^7^. Animals were randomly assigned to the treatment groups, pseudonymized by an independent investigator. The technician responsible for echocardiographic evaluation and the person interpreting the electrocardiograms were blinded to the group assignment. The investigation conforms to the guide for the care and use of laboratory animals published by the NIH (Publication No. 85–23, revised 1985) and was approved by the local authorities (Behörde für Gesundheit und Verbraucherschutz, Freie und Hansestadt Hamburg: 61/15).

### Differentiation of human cardiomyocytes

Human cardiomyocytes were generated as previously described^20^. Human induced pluripotent stem cells (hiPSCs) were maintained on Geltrex^®^ (Gibco A1413302) in FTDA (DMEM/F-12 (Gibco 21331–046), 2 mM L-glutamine (Gibco 25030-081), 0.1% (vol/vol) lipid mix (Sigma-Aldrich L5146), 5 mg/L transferrin (Sigma-Aldrich T8158), 5 µg/L selenium (Sigma-Aldrich S5261), 0.1% (vol/vol) human serum albumin (Biological Industries 05-720-1B), 5 µg/ml insulin (Sigma-Aldrich I9278), 30 ng/ml BMP-4 (R&D Systems 314-BP), 2.5 ng/ml Activin-A (R&D Systems 338-AC), 50 nM Dorsomorphin (abcam ab120843) and 0.5 ng/ml TGF-β1 (Peprotech 100-21). For embryoid body (EB) formation, the hiPSCs were dissociated into single cells (0.5 mM EDTA for 10 min at 37 °C), re-suspended in FTDA containing 4 mg/ml PVA (Sigma-Aldrich P8136) and 10 µM Y-27632 (biorbyt orb60104), seeded into spinner flasks (Integra 182 051, 182 101) at 30 × 10^6^ cells/100 ml and rotated overnight at 40 rounds per minute, 37 °C, 90% humidity, 5% CO_2_, 5% O_2_. For induction of mesodermal progenitor cells, EBs were cultivated in Pluronic F-127 (Sigma-Aldrich, P2443) -coated cell culture flasks in mesoderm induction medium (RPMI 1640, Gibco 21875; 4 mg/ml PVA, Sigma-Aldrich P8136; 10 mM HEPES, Roth 9105.4; 0.05% (vol/vol) human serum albumin, Biological Industries 05-720-1B; 0.1% lipid mix, Sigma-Aldrich L5146; 250 µM phosphoascorbat, Sigma-Aldrich 49752; 5 mg/L transferrin, Sigma-Aldrich T8158; 5 µg/L selenium, Sigma-Aldrich S5261; 0.5% penicillin/streptomycin, Gibco 15140; 10 µM Y-27632, biorbyt orb60104; 10 ng/ml BMP-4, R&D Systems 314-BP; 3 ng/ml Activin-A, R&D Systems 338-AC; 5 ng/ml basic FGF, R&D Systems 233-FB) for 3 days at 37 °C, 90% humidity, 5% CO_2_, 5% O_2_ with daily medium change. For cardiac differentiation, EBs were transferred to Cardiac Differentiation Medium I (RPMI 1640, Gibco 21875; 10 mM HEPES, Roth 9105.4; 0.05% (vol/vol) human serum albumin, Biological Industries 05-720-1B; 0.1% lipid mix, Sigma-Aldrich L5146; 250 µM phosphoascorbat, Sigma-Aldrich 49752; 5 mg/L transferrin, Sigma-Aldrich T8158; 5 µg/L selenium, Sigma-Aldrich S5261; 0.5% penicillin/streptomycin, Gibco 15140; 1 µM Y-27632, biorbyt orb60104; 100 nM 4-(*cis-endo*-1,3-dioxooctahydro-*2H*-4,7-methanoisoindol-2-yl)-*N*-(quinolin-8-yl)-*trans*-cyclohexylcarboxamide (DS-I-7)) and cultivated in Geltrex®-coated cell culture flasks for 3 days at 37 °C, 90% humidity, 5% CO_2_, 21% O_2_ with daily medium change. On day 7 medium was changed to Cardiac Differentiation Medium II (RPMI 1640, Gibco 21875; 500 µM 1-Thioglycerol, Sigma-Aldrich M6145; 10 mM HEPES, Roth, 9105.4; 0.5% penicillin/streptomycin, Gibco, 15140; 1 µM Y-27632, biorbyt orb60104; 2% (vol/vol) B27 plus insulin, Gibco, 17504-044; 1 μM XAV 939, Tocris, 3748). EBs were incubated for additional 4 days with daily media change under standard culture conditions. From day 11 to 14 EB culture was extended without Wnt-signalling inhibitor.

### Differentiation of human endothelial cells

Human endothelial cells were generated as previously described^7^. Maintenance and formation of EBs was performed as described in the paragraph cardiac differentiation. For induction of differentiation of mesodermal progenitor cells, EBs were cultivated in Pluronic F-127 (Sigma-Aldrich, P2443) -coated flasks in StemPro^®^-34 (Gibco 10639-011) supplemented with 4 mg/ml PVA; 400 µM 1-thioglycerol, Sigma-Aldrich M6145; 2 mM L-glutamin, Gibco 25030; 5 mg/L transferrin, Sigma-Aldrich T8158; 5 µg/L selenium, Sigma-Aldrich S5261; 0.5% Penicillin/Streptomycin, Gibco 15140; 10 µM Y-27632, biorbyt orb60104 and 10 ng/ml BMP-4, R&D Systems 314-BP; 6 ng/ml Activin-A, R&D systems 338-AC; 5 ng/ml basic FGF, R&D systems 233-FB for 3 days at 37 °C, 90% humidity, 5% CO_2_, 5% O_2_ with daily medium change. To differentiate endothelial cells, EBs were then cultured on Geltrex^®^ in StemPro^®^-34, containing 400 µM 1-Thioglycerol, Sigma-Aldrich M6145; 2 mM L-Glutamin, Gibco 25030; 1 mM magnesium ascorbyl phosphate; 5 mg/L transferrin, Sigma-Aldrich T8158; 5 µg/L selenium, Sigma-Aldrich S52610.5% penicillin/streptomycin, Gibco 15140; 1 µM Y-27632, biorbyt orb60104; 100 ng/ml VEGF, R&D Systems 293-VE and 10 ng/ml bFGF, R&D Systems 233-FB. For 3 days, EBs were cultured in a hypoxic environment (5% CO_2_, 5% O_2_), followed by 9 days under normoxic conditions. Medium was changed every other day.

### Magnetic cell sorting of hiPS-endothelial cells

On day 14 of differentiation, adherent EBs were dissociated to single-cell suspensions with collagenase 2 (200 U/ml, Worthington LS004176) in HBSS for 1.5 h. For MACS, cells were incubated with CD31-antibodies conjugated to magnetic beads (Miltenyi Biotech 130-091-935) and sorted twice on LS columns (Miltenyi Biotech 130-042-401) according to the manufacturer’s protocol to 95% purity. Sorted CD31-positive cells were cultivated on 1% gelatin in human endothelial growth (serum free) medium (LifeTechnologies 11111) supplemented with 1% platelet poor plasma (BTI BT-214), 30 ng/ml VEGF (R&D Systems, 293-VE) and 20 ng/ml bFGF (R&D systems 233-FB).

### hEHT generation and cell free graft generation

HEHTs were generated as previously described^20,28^. HiPS-cardiomyocytes were dissociated to single-cell suspensions with collagenase 2 (200 U/ml, Worthington, LS004176 in HBSS minus Ca^2+^/Mg^2+^, Gibco, 14175-053) for 3.5 h and MACS-sorted hiPS-endothelial cells were harvested with TrypLE select (5 min., 37 °C, Gibco 12563-029) and resuspended in DMEM (Biochrom, F0415) supplemented with 10% (vol/vol) heat-inactivated FCS, (Biochrom S0615), 1% (vol/vol) Glutamin (Gibco 25030-081) and 1% (vol/vol) Penicillin/Streptomycin (Gibco 15140). hEHTs were generated in 6-well format and contained 5 × 10^6^ hiPS-cardiomyocytes and 2 × 10^6^ hiPS-endothelial cells per hEHT. The EHT mastermix consisted of 80% cell suspension, 10% (vol/vol) Matrigel, BD 354234; 5.5% (vol/vol) 2× DMEM (20% (vol/vol) 10× DMEM, Gibco 52100-021; 20% (vol/vol) heat-inactivated Horse Serum, life technologies 26050088; 2% (vol/vol) Penicillin/Streptomycin, Gibco 15140; 58% (vol/vol) water) and 5 mg/ml fibrinogen (Sigma-Aldrich F8630). For each hEHT, 700 μL fibrinogen containing mastermix was mixed briefly with 21 μl thrombin (100 U/mL, Sigma Aldrich T7513) and pipetted into the agarose slot. Cell-free grafts were produced in the same manner as hEHTs, however did not contain cardiomyocytes and endothelial cells. Human EHTs and cell free constructs were cultivated in DMEM (Gibco 41965-039) supplemented with 1% Penicillin/Streptomycin (vol/vol), Gibco 15140; 10% heat-inactivated Horse Serum, Gibco 26050; 0.1% Insulin, 10 µg/ml, Sigma I9278; 0.1% Aprotinin, 33 µg/ml, Sigma A1153. Spacers and silicone racks for 6-well format EHTs were hand-made.

### ECG transmitter implantation and cryo-injury model

ECG transmitters were implanted during the same procedure when the myocardial cryo-injury was performed (day 0, Fig. 1a). Female guinea pigs at the age of 4 to 6 weeks (280–370 g) were anesthesized with isoflurane (2.5%) and were placed on a warming platform in a supine position. Carprofen (5 mg/kg body weight) and buprenorphine (0.05 mg/kg body weight) were injected subcutaneously 30 min prior to the procedure. The skin was shaved, and a 1 cm incision was made in the right flank. A transmitter (PhysioTel^TM^ ETA-F10, D.S.I. Data Sciences International, MN, USA) was inserted in a subcutaneous pocket and fixed with a 5-0 prolene suture. The positive ECG-lead was tunneled to the fifth left intercostal space and the negative lead was tunneled and fixed 1 cm lateral to the upper sternal midline. The animal was then intubated and mechanically ventilated. Left lateral thoracotomy was performed, and the pericardium was opened. Cryo-injury of the left ventricular wall was induced with an aluminum probe (diameter 0.5 cm) pre-cooled in liquid nitrogen, which was applied 4 times for 30 seconds each^7,9^.

### hEHT-transplantation

hEHTs or cell-free grafts were transplanted onto the guinea pig hearts 7 days after cryo-injury^7^. Constructs were sutured onto the healthy myocardium adjacent to the scar. Guinea pigs were immunosuppressed with methylprednisolone (2 mg/kg body weight/day) and ciclosporin (5 mg/kg body weight/day). Animals received buprenorphine (0.1 mg/kg body weight/day) and carprofen (5 mg/kg body weight/day) for 5 postoperative days.

### Histology

Histological analysis was performed as recently described by our group^7^. Hearts were harvested, fixed in neutral buffered 4% formaldehyde/1% methanol, pH 7.4 and sectioned in three transverse sections (apical, mid-papillary and basal). These sections were further processed for paraffin embedding. Microscopic images were taken on an Axioskop 2 microscope (Zeiss, Jena, Germany).

### ECG recordings and analysis

Following implantation of the devices, ECGs were recorded over a period of 35 days (Fig. 1a). During recordings, animals were housed in individual cages on a receiver plate and allowed free access to food and water. Day-night rhythm was established with daytime (lights on) between 07:00 AM–07:00 PM. Room temperature and humidity were controlled at 20–22 °C and 50–70%, respectively. ECG recordings were performed at a sampling rate of 1 kHz using Dataquest A.R.T. (v 4.3, D.S.I.) and PONEMAH (v 5.2, D.S.I.). ECG waveforms and ECG parameters were stored for one minute every five minutes.

Offline ECG analysis was performed using Dataquest A.R.T. (v. 4.3, D.S.I.) and Ponemah (v 6.0, D.S.I.) by an operator blinded to the experimental groups. Abnormal ventricular contractions were identified based on irregularities in consecutive RR intervals consistent with ventricular arrhythmias (e.g. premature contractions, post-extrasystolic pause). Arrhythmias were classified as previously, based on the guidelines of The Lambeth Conventions^29,30^. Premature ventricular contractions (PVC) were identified by the presence of at least two of the following three criteria:

1. atypical QRS complex or QRS vector
2. absence of detectable P-wave or atrioventricular dissociation
3. abbreviated RR interval before and compensatory pause following the beat

Two or three consecutive PVCs were defined as couplets or triplets, respectively. A run of four or more consecutive PVCs (≤15 PVCs) was defined as non-sustained ventricular tachycardia (nsVT), whereas a sustained VT (susVT) consisted of ≥16 consecutive PVCs. Ventricular fibrillation (VF) was identified by the absence of distinguishable individual QRS complexes and of isoelectric phases. Controversial ECG segments were classified and consented by three independent ECG experts.

To avoid analysis of postoperative artefacts, e.g. due to anesthetic drugs, ECGs were analyzed starting 48 h after the cryo-injury and the hEHT implantation procedure, respectively. The direct perioperative periods were excluded a priori.

### Echocardiography

Transthoracic echocardiography was performed using a Vevo 2100 system (VisualSonics, Toronto, Canada). Animals were anesthetized with isoflurane (1.5–2%) and placed on a warming platform in a supine position. Two-dimensional short axis views were recorded at the mid-papillary level. Parasternal long axis views were recorded at the plane of the aortic valve with a concurrent visualization of the left-ventricular apex. Conventional measurements were obtained from B-mode recordings using a MS 400 transducer (center frequency 30 MHz) with a frame rate of 230–400 frames/s. Anterior and posterior wall thickness, left-ventricular diameter and the area of the left-ventricular cavity were recorded according to standard procedures. All images were recorded digitally, and off-line analysis was performed using the Vevo 2100 software.

### Statistical analysis and data availability

Data are given as indicated. Statistical analysis was performed using Graphpad Prism (v. 6.0, GraphPad Software Inc., San Diego, CA, USA) and Stata 15.1 (StataCorp LLC, Texas, USA). Statistical significance was calculated as indicated in the figure legends. Statistical significance was defined as P < 0.05. The datasets generated during and/or analyzed during the current study are available from the corresponding author on reasonable request.
